# Thyroid Hormone Is Related to Postoperative AKI in Acute Type A Aortic Dissection

**DOI:** 10.3389/fendo.2020.588149

**Published:** 2020-11-18

**Authors:** Jihong Liu, Yuan Xue, Wenjian Jiang, Hongjia Zhang, Yuanfei Zhao

**Affiliations:** ^1^ Department of Cardiac Surgery, Beijing Anzhen Hospital, Capital Medical University, Beijing, China; ^2^ Beijing Institute of Heart, Lung and Blood Vessel Diseases, Beijing, China; ^3^ Beijing Lab for Cardiovascular Precision Medicine, Beijing, China; ^4^ Department of Cardiac Surgery, Affiliated Zhongshan Hospital of Dalian University, Dalian, China; ^5^ Centre for Transplant and Renal Research, The Westmead Institute for Medical Research, University of Sydney, Sydney, NSW, Australia

**Keywords:** thyroid hormones, acute kidney injury, surgery, postoperative, aortic dissection

## Abstract

**Background:**

Renal function is profoundly influenced by thyroid hormone levels. This study was designed to evaluate the association between preoperative thyroid hormones and postoperative acute kidney injury (AKI) in acute type A aortic dissection (ATAAD) patients.

**Methods:**

A total of 88 patients with ATAAD who underwent surgeries in Beijing Anzhen Hospital and 274 healthy controls from July 2016 to December 2016 were included in this study. Propensity-score matching was used to compare thyroid hormone levels. Additionally, in a cohort study of ATAAD patients, multivariable regression and stratification analyses were conducted to examine the association of preoperative thyroid hormones with postoperative AKI.

**Results:**

Compared with healthy controls, ATAAD patients presented with lower preoperative levels of total triiodothyronine (TT3) (P < 0.01), free triiodothyronine (FT3) (P < 0.01), and thyroid-stimulating hormone (TSH) (P < 0.01) and a higher preoperative level of free thyroxine (FT4) (P < 0.01). The overall occurrence of postoperative AKI was 45.5%. Multivariate regression revealed that low levels of TT3 (OR = 0.07, 95% CI, 0.01–0.86, P = 0.04) were independently associated with postoperative AKI. Subgroup analyses showed that the association between TT3 and AKI was significant in patients with normal TSH levels (OR = 0.001 95% CI, 0.001–0.16, P < 0.01) but not in patients with lower TSH levels (P = 0.12).

**Conclusion:**

The present study showed that a low level of TT3 was a predictor of postoperative AKI in ATAAD patients, especially in patients with normal TSH. The thyroid function should be checked before surgical intervention of patients with ATAAD, and patients with low T3 might be at higher risk of postoperative AKI.

## Introduction

Thyroid hormone abnormalities are common phenomena in patients with non-thyroidal illnesses, including cancer, infectious diseases, burns, and trauma. This condition is also called non-thyroidal illness syndrome (NTIS) (1, 2) or euthyroid sick syndrome (ESS) (3). NTIS consists of a low level of circulating values of triiodothyronine (T3) and normal or low levels of thyroxine (T4) and thyroid-stimulating hormone (TSH) (4). This phenomenon was first reported in patients admitted to intensive care units, and it was also observed in various critical conditions, such as acute myocardial infarction (5), heart failure (6), and in the postoperative period of cardiac surgery (7). Low levels of T3 were observed in 15–30% of patients with heart failure (6, 8) and in 15–20% of patients with acute myocardial infarction (5, 9). Critical illness-induced NTIS is characterized by decreases in T3, circulating T4, and TSH (4). Severe illness induces dramatic changes in thyroid metabolism, resulting in a downregulation of the hypothalamic–pituitary–thyroid axis at both the hypothalamic and pituitary levels, with related decreases in circulating thyroid hormone concentrations (10). Under such conditions, changes in thyroid function are associated with adverse outcomes, which include acute kidney injury (AKI) (11).

Acute type A aortic dissection (ATAAD) is a life-threatening disorder. Urgent surgical intervention is recommended according to the current guidelines (12), while AKI is a common postoperative complication (13). It has been acknowledged that age and clinical presentation as well as the technique and duration of cardiopulmonary bypass are associated with developing AKI in ATAAD (14, 15). The thyroid hormones play significant roles in the growth, development, and physiology of the kidney (16). However, the association between preoperative thyroid hormone and postoperative AKI has not been investigated, even with an increasing number of studies showing the effects of thyroid metabolism on renal function (17, 18). This study was designed to analyze the association between preoperative thyroid hormones and postoperative AKI in ATAAD patients.

## Methods

### Patient Selection

A total of 88 patients with ATAAD who underwent surgeries in Beijing Anzhen Hospital and were enrolled in “*A Study of the Prediction and Treatment of Acute Aortic Syndrome (ChiCTR1900022637)*” from July 2016 to December 2016 were included for the purpose of this analysis. Patients who had AKI, a history of thyroid disease, a history of pituitary diseases, or a history of administration of medicine that affects thyroid function were excluded. Patients with disease that affects thyroid binding protein concentration, such as dysfunction of liver, pregnancy, and malignant tumors, were also excluded. Patients who died within 24 h after surgery were also excluded because the data were inappropriate for the evaluation of postoperative renal function. In addition, 243 healthy people without a history of thyroid functional abnormality were included as the controls of the thyroid hormone profile.

The distribution of surgical procedures was as follows: 45 patients received Bentall procedure (including one patient with CABG, 25 patients with total arch replacement using a tetrafurcate graft and stented elephant trunk implantation (19), three patients with total arch replacement using a tetrafurcate graft and stented elephant trunk implantation and CABG, and two patients with total arch replacement using a tetrafurcate graft and stented elephant trunk implantation and tricuspid valve repair), and 43 patients received ascending aortic replacement (including 22 patients with total arch replacement using a tetrafurcate graft and stented elephant trunk implantation and one patient with total arch replacement using a tetrafurcate graft and stented elephant trunk implantation and CABG). The right axillary artery was used for antegrade selective cerebral perfusion when performing total arch replacement using a tetrafurcate graft and stented elephant trunk implantation under moderate hypothermia circulation arrest.

### Data Collection

Demographic variables included sex, age, body mass index (BMI), and comorbidities (hypertension, smoking, diabetes, and cardiovascular disease). Laboratory data included preoperative serum creatinine level, platelet (PLT) counts, red blood cell (RBC) counts, hematocrit (HCT) levels and preoperative thyroid function parameters. Intraoperative data included the time of cardiopulmonary bypass (CPB) and aortic cross-clamping, nasopharyngeal temperature (the lowest temperature recorded during CPB), moderate hypothermic circulatory arrest, circulatory arrest time and intraoperative packed red blood cell (PRBC) transfusion. Postoperative variables included intensive care unit (ICU) retention time and renal function.

### Thyroid Hormone Sampling

The thyroid function profiles of all patients were detected before surgery. Blood samples were collected and immediately centrifuged and analyzed. In the present study, free triiodothyronine (FT3), total triiodothyronine (TT3), free thyroxine (FT4), total thyroxine (TT4), and thyroid-stimulating hormone (TSH) were measured in all samples with Access thyroid function kits (Beckman counter corporation, chemiluminescent immunoassay, Access Total T3 kit 33830, Access Total T4 kit 33800, Access HYPER sensitive hTSH kit 33820, Access Free T3 kit A13422 and Access Free T4 33880).

### Definitions and End Point

In this study, ATAAD was considered a dissection that involved the ascending aorta with presentation of symptom onset less than 14 days according to the guideline (12). The primary clinical end point was postoperative AKI. AKI was defined according to the newest consensus-based KDIGO criteria as follows (20): small changes in serum creatinine (≥0.3 mg/dl or 26.5 μmol/l) when they occurred within 48 h or a maximal change in serum creatinine ≥1.5 times the baseline value until postoperative day 7 compared with preoperative baseline values or urine volume <0.5 ml/kg/h for 6 h. In this study, we did not take urine output into consideration because of its inaccuracy.

### Statistical Analysis

Given the differences in the baseline characteristics between the healthy group and the ATAAD group (**Table 1**), propensity-score (PS) matching was used to identify participants with similar baseline characteristics. ATAAD patients were matched 1:1 to healthy people by their PSs using the nearest neighbor method with a caliper of 0.05 and no replacement. The PS was obtained by the use of a multivariate logistic regression model and six baseline characteristics (age, sex, BMI, hypertension, diabetes, and smoking) as covariates.

**Table 1:** Baseline characteristics before and after propensity-score matching.

Variables	Before matching			After matching			P value
	Control N = 274	ATAAD N = 88	P value	Control N = 83	ATAAD N = 83	Standardized diff.	
Age (years), mean ± SD	48.2 ± 11.9	50.7 ± 11.2	0.08	51.1 ± 12.1	50.8 ± 11.3	0.02	0.88
BMI (kg/m^2^), mean ± SD	24.7 ± 3.9	25.9 ± 3.7	0.01*	26.0 ± 3.2	25.7 ± 3.5	0.09	0.58
Sex (Female), n (%)	72 (26.3%)	31 (35.2%)	0.11	30 (36.1%)	31 (37.3%)	0.03	1.00
Hypertension, n (%)	47 (17.3%)	44 (50%)	<0.01*	39 (47%)	39 (47%)	0	1.00
Diabetes, n (%)	20 (7.4%)	4 (4.5%)	0.36	4 (4.8%)	4 (4.8%)	0	1.00
Smoking, n (%)	89 (32.8%)	18 (20.5%)	0.03*	19 (22.9%)	17 (20.5%)	0.06	0.85
TT3 (nmol/L), mean ± SD	1.5 ± 0.5	0.8 ± 0.3	<0.01*	1.5 ± 0.3	0.8 ± 0.3	2.45	<0.01*
TT4 (nmol/L), mean ± SD	100.5 ± 19.4	96.9 ± 22.0	0.15	100.1 ± 21.1	97.5 ± 22.6	0.12	0.44
TSH (mIU/L), mean ± SD)	2.2 ± 1.5	1.2 ± 1.1	<0.01*	2.0 ± 1.1	1.3 ± 1.1	0.73	<0.01*
FT3 (pmol/L), mean ± SD	5.3 ± 1.7	3.7 ± 0.8	<0.01*	5.2 ± 0.7	3.7 ± 0.8	2.00	<0.01*
FT4 (pmol/L), mean ± SD	11.6 ± 5.6	13.3 ± 2.6	<0.01*	11.1 ± 1.5	13.3 ± 2.6	1.07	<0.01*

Results are expressed as n (%) or mean ± standard deviation (SD). The standardized differences are reported as percentages; a difference of less than 10.0% indicates a relatively small imbalance.

BMI, body mass index; FT3, free triiodothyronine; TT3, total triiodothyronine; FT4, free thyroxine; TT4, total thyroxine; TSH, thyroid stimulating hormone; ATAAD, acute type A aortic dissection.

*P value indicates significance at P < 0.05.

In this cohort study of 88 ATAAD patients, continuous variables are presented as the means ± SDs or medians (25th–75th percentiles), and categorical data are represented as frequencies and percentages. The two-tailed Student’s t test or Wilcoxon rank-sum test was applied to compare continuous variables, whereas the chi-square test or Fisher test was used for comparing categorical variables. Logistic regression analysis was performed to identify the predictors of postoperative AKI. Multivariable regression was used to assess the association between preoperative thyroid function and the postoperative AKI. Based on the recommendations of the STROBE statement, we also showed the results of the unadjusted, minimally adjusted and fully adjusted analyses. We selected these confounders on the basis of their associations with the outcomes of interest or the changes of the effect estimate of more than 10% (21). In addition, the generalized additive model was used to assess the non-linear relationships. In the subgroup analyses, the clinical cutoffs of TSH were used (18), and two categories were defined: TSH ≤0.49 mIU/L as the low TSH group and TSH >0.49 mIU/L as the normal TSH group (all patients’ TSH levels were less than 4.91 mIU/L). We conducted stratification analyses to examine whether the effects of the levels of the thyroid hormones differed across various subgroups classified by TSH level, and their interactions were tested. A two-tailed P value <0.05 was considered statistically significant. All of the analyses were performed with the statistical software packages R (http://www.R-project.org, The R Foundation) and EmpowerStats (http://www.empowerstats.com, X&Y Solution, Inc., Boston, MA).

## Results

### Propensity-Score Matching of ATAAD Patients and Healthy Controls

A total of 88 patients with ATAAD who underwent surgeries in Beijing Anzhen Hospital, and 274 healthy controls from January 2016 to January 2017 were included in this study (**Table 1**). After PS matching, 83 patients with ATAAD were matched with 83 healthy people. The standardized differences were less than 10% for all covariates. Compared with healthy people, patients with ATAAD had lower preoperative levels of TT3 (0.8 ± 0.3 *vs* 1.5 ± 0.3, P < 0.01), FT3 (3.7 ± 0.8 *vs* 5.2 ± 0.7, P < 0.01) and TSH (1.3 ± 1.1 *vs* 2.0 ± 1.1, P < 0.01) and a higher preoperative level of FT4 (13.3 ± 2.6 *vs* 13.3 ± 2.6, P < 0.01).

### Characteristics of the Patients

The clinical characteristics of ATAAD patients are shown in **Table 2**. The mean age was 50.7 ± 11.2 years, and 57 (64.8%) patients were male. Medical histories included hypertension (50%), smoking (20.5%), diabetes (4.5%), and previous cardiovascular disease (5.7%). The mean CPB time was 212.1 ± 69.2 min, and the mean time of aortic cross-clamping was 117.3 ± 38.6 min. Moderate hypothermic circulatory arrest was used in 81 patients (92%) with mean time of 23.0 ± 7.7 min. The average nasopharyngeal temperature was 24.2 ± 1.8°C. Intraoperative PRBC transfusion was used in 48 patients (54.6%), with a median level of two units (range, 0 to 10 units). The mean preoperative creatinine level was 84.0 ± 38.9 µmol/L, and 40 patients (45.5%) had postoperative AKI.

**Table 2:** Baseline characteristics of participants.

Variables	N = 88
**Demographics**	
Age(years), mean ± SD	50.7 ± 11.2
BMI (kg/m^2^), mean ± SD	25.9 ± 3.7
Sex (Female), n (%)	31 (35.2%)
**Comorbidities**	
Hypertension, n (%)	44 (50.0%)
Smoking, n (%)	18 (20.5%)
Diabetes, n (%)	4 (4.5%)
Cardiovascular disease, n (%)	5 (5.7%)
**Laboratory values**	
TT3 (nmol/L), mean ± SD	0.8 ± 0.3
TT4 (nmol/L), mean ± SD	96.9 ± 22.0
TSH (mIU/L), mean ± SD	1.2 ± 1.1
FT3 (pmol/L), mean ± SD	3.7 ± 0.8
FT4 (pmol/L), mean ± SD	13.3 ± 2.6
Creatinine (μmol/L), mean ± SD	84.0 ± 38.9
Platelet (G/L), mean ± SD	122.0 ± 86.2
Red blood cell (×10^12^), mean ± SD	3.6 ± 0.6
Hematocrit (%), mean ± SD	38.3 ± 5.6
Ejection fraction (%), mean ± SD	61.7 ± 5.2
Pericardial effusion, n (%)	18 (22.8%)
**Intraoperative data**	
Nasopharyngeal temperature (℃), mean ± SD	24.2 ± 1.8
Intraoperative transfusion of PRBCs (u), median (IQR)	2.0 (0.0–4.0)
CPB time (min), mean ± SD	212.1 ± 69.2
Aortic cross-clamping time (min), mean ± SD	117.3 ± 38.6
Moderate hypothermic circulatory arrest, n (%)	81 (92.0%)
Circulatory arrest time (min), mean ± SD	23.0 ± 7.7
**Postoperative outcome**	
ICU retention time (day), median (IQR)	1.86 (0.94–3.51)
AKI, n (%)	40 (45.5%)

Results are expressed as n (%) or mean ± standard deviation (SD) or median interquartile range (IQR).

BMI, body mass index; FT3, free triiodothyronine; TT3, total triiodothyronine; FT4, free thyroxine; TT4, total thyroxine; TSH, thyroid stimulating hormone; PRBC, packed red blood cell; CPB, cardiopulmonary bypass; ICU, intensive care unit; AKI, acute kidney injury.

### Univariate Analysis and Multivariate Analyses

The results of univariate analysis (**Table 3**) indicated that age (OR = 1.05, 95% CI, 1.01–1.09, P = 0.03) and intraoperative transfusion of PRBCs (OR = 1.31, 95% CI, 1.09–1.57, P < 0.01) were significantly correlated with postoperative AKI. However, thyroid hormone levels were not associated with postoperative AKI. After adjustments for age, sex, BMI, PLT, intraoperative transfusion of PRBCs, HCT, creatinine, circulatory arrest time, and aortic cross-clamping time, preoperative TT3 level (OR = 0.07, 95% CI, 0.01–0.86, P = 0.04) was independently associated with AKI (**Table 4**).

**Table 3:** Univariate analysis of risk factors associated with postoperative AKI in patients with ATAAD.

Variables	OR (95%CI)	P-value
Age (years)	1.05 (1.01, 1.09)	0.03*
BMI (kg/m^2^)	1.02 (0.91, 1.15)	0.72
Sex (Female)	1.50 (0.61, 3.68)	0.38
Hypertension	0.95 (0.40, 2.23)	0.90
Smoking	1.05 (0.37, 2.99)	0.93
Diabetes	4.15 (0.41, 41.61)	0.23
Cardiovascular disease	2.03 (0.32, 12.82)	0.45
TT3 (nmol/L)	0.27 (0.04, 1.69)	0.16
TT4 (nmol/L)	0.98 (0.96, 1.01)	0.14
TSH (mIU/L)	1.06 (0.71, 1.59)	0.76
FT3 (pmol/L)	0.71 (0.39, 1.30)	0.27
FT4 (pmol/L)	0.98 (0.83, 1.16)	0.82
Creatinine (μmol/L)	0.99 (0.98, 1.01)	0.20
Platelet (G/L)	0.99 (0.99, 1.00)	0.07
Red blood cell (×10^12^)	0.63 (0.31, 1.28)	0.20
Hematocrit (%)	1.00 (0.92, 1.08)	0.93
Ejection fraction (%)	1.09 (0.99, 1.20)	0.08
Pericardial effusion	0.56 (0.17, 1.80)	0.33
Intraoperative transfusion of PRBCs (u)	1.31 (1.09, 1.57)	<0.01*
CPB time (min)	1.00 (0.99, 1.00)	0.23
Aortic cross-clamping time (min)	0.99 (0.98, 1.00)	0.11
Moderate hypothermic circulatory arrest	0.28 (0.05, 1.52)	0.14
Circulatory arrest time (min)	0.96 (0.91, 1.01)	0.08
Nasopharyngeal temperature (℃)	0.83 (0.63, 1.10)	0.19

ATAAD, acute type aortic dissection; BMI, body mass index; FT3, free triiodothyronine; TT3, total triiodothyronine; FT4, free thyroxine; TT4, total thyroxine; TSH, thyroid stimulating hormone; PRBC, packed red blood cell; CPB, cardiopulmonary bypass; AKI, acute kidney injury.

*P value indicates significance at P < 0.05.

**Table 4:** Multivariable analysis to assess the independent impact of thyroid hormone on AKI in patients with ASTAAD using none adjusted and adjusted logistic regression model.

	Crude Model		Model Ⅰ		Model ⅠⅠ	
	OR(95%CI)	P-value	OR(95%CI)	P-value	OR(95%CI)	P-value
TT3 (nmol/L)	0.27 (0.04, 1.69)	0.16	0.32 (0.05, 2.23)	0.25	0.07 (0.01, 0.86)	0.04*
TT4 (nmol/L)	0.98 (0.96, 1.01)	0.14	0.98 (0.96, 1.01)	0.17	0.97 (0.94, 1.00)	0.07
TSH (mIU/L)	1.06 (0.71, 1.59)	0.76	1.03 (0.66, 1.61)	0.89	1.00 (0.61, 1.64)	0.98
FT3 (pmol/L)	0.71 (0.39, 1.30)	0.27	0.88 (0.45, 1.70)	0.69	0.71 (0.30, 1.66)	0.43
FT4 (pmol/L)	0.98 (0.83, 1.16)	0.82	0.97 (0.81, 1.15)	0.69	0.96 (0.78, 1.19)	0.72

Crude Model adjusted for: None.

Model I adjusted for: sex; age (years); body mass index (kg/m^2^)

Model II adjusted for: sex; age (years); body mass index (kg/m^2^); platelet (G/L); Hematocrit (%); creatinine (μmol/L); intraoperative transfusion of packed red blood cell (u); aortic cross-clamping time (min); circulatory arrest time (min).

*P value indicates significance at P < 0.05.

### Subgroup Analyses

Subgroup analyses (**Table 5**) stratified by preoperative TSH levels showed that the association between TT3 and AKI remained significant in patients with normal preoperative TSH levels in all models, but not in patients with lower preoperative TSH levels. Additionally, interaction analyses found significant interactions between TSH and TT3 or TSH and FT3 with regard to the effect on the risk of AKI (P < 0.01 and P = 0.01, respectively).

**Table 5:** Subgroup analysis of the associations between AKI and the thyroid hormone.

	Crude Model		Model Ⅰ		Model ⅠⅠ		P for interaction
	OR(95%CI)	P-value	OR(95%CI)	P-value	OR(95%CI)	P-value	
TT3 (nmol/L)							<0.01*
Normal TSH	0.04 (0.004, 0.45)	<0.01*	0.04 (0.004, 0.55)	0.02*	0.001 (0.001, 0.16)	<0.01*	
Lower TSH	46.95 (0.61, 3632.43)	0.08	203.57(1.12, 36874.33)	0.06	150.69 (0.29, 77935.173)	0.12	
FT3 (pmol/L)							0.01*
Normal TSH	0.43(0.18, 0.99)	0.05	0.57 (0.23, 1.38)	0.21	0.22 (0.05, 1.07)	0.06	
Lower TSH	1.86(0.56, 6.20)	0.31	2.22 (0.61, 8.00)	0.22	1.79 (0.24, 13.42)	0.57	
TT4 (nmol/L)							0.67
Normal TSH	0.98 (0.95, 1.00)	0.10	0.98 (0.96, 1.01)	0.28	0.97 (0.93, 1.01)	0.14	
Lower TSH	0.99 (0.96, 1.04)	0.84	0.99 (0.95, 1.04)	0.63	0.95 (0.87, 1.04)	0.27	
FT4 (pmol/L)							0.20
Normal TSH	1.00 (0.81, 1.23)	0.99	0.99(0.79, 1.23)	0.89	1.08 (0.79, 1.49)	0.62	
Lower TSH	0.93 (0.69, 1.26)	0.65	0.95(0.69, 1.31)	0.74	0.76 (0.47, 1.23)	0.26	

Crude model adjusted for: None.

Model I adjusted for: sex; age (years); body mass index (kg/m^2^).

Model II adjusted for: sex; age (years); body mass index (kg/m^2^); platelet (G/L); Hematocrit (%); creatinine (μmol/L); intraoperative transfusion of packed red blood cell (u); aortic cross-clamping time (min); circulatory arrest time (min).

*P value indicates significance at P < 0.05.

### Non-Linear Relationships

**Figure 1:**
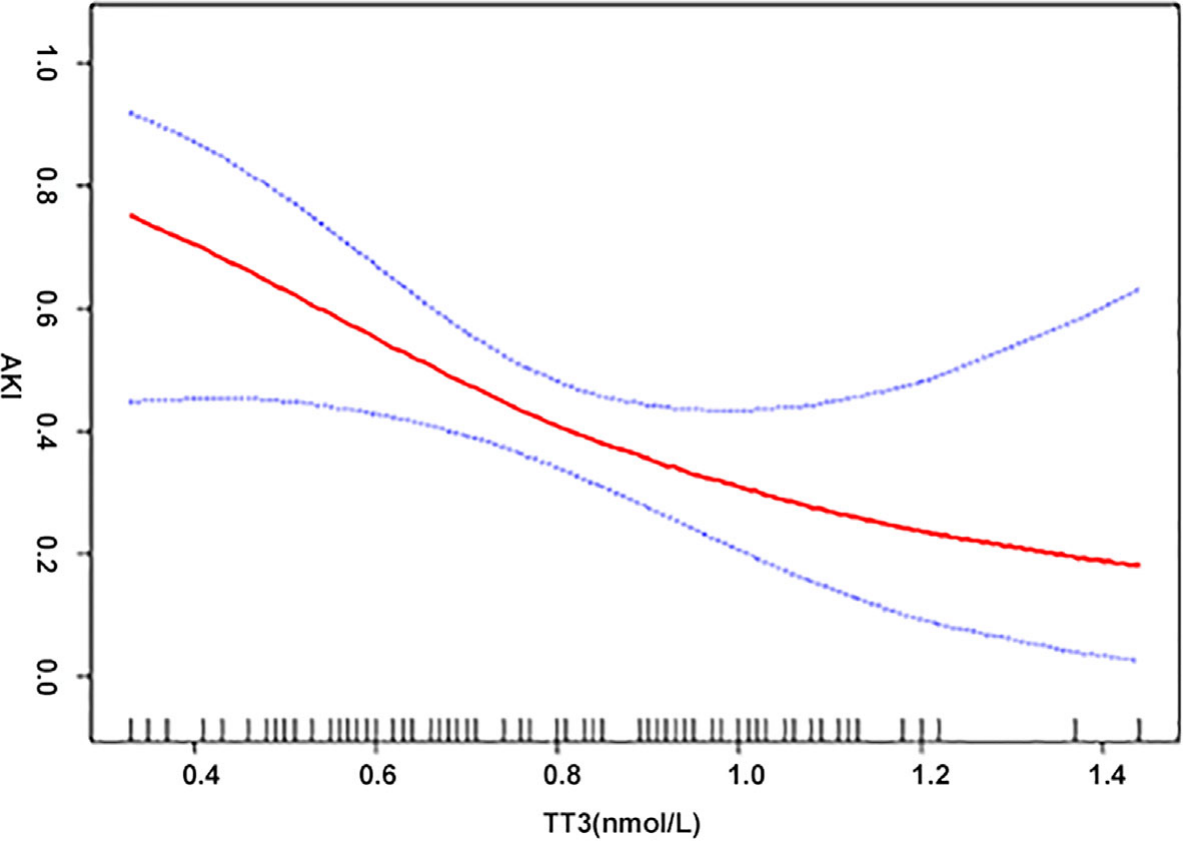
The relationship between thyroid hormone and AKI. A non-linear association between TT3, TT4, and AKI was found in a generalized additive model (GAM). Solid red line represents the smooth curve fit between variables. Blue bands represent the 95% of CI from the fit. Adjusted for age (years); body mass index (kg/m^2^); platelet (G/L); hematocrit (%); creatinine (μmol/L); intraoperative transfusion of packed red blood cell (u); aortic cross-clamping time (min).

**Figure 2:**
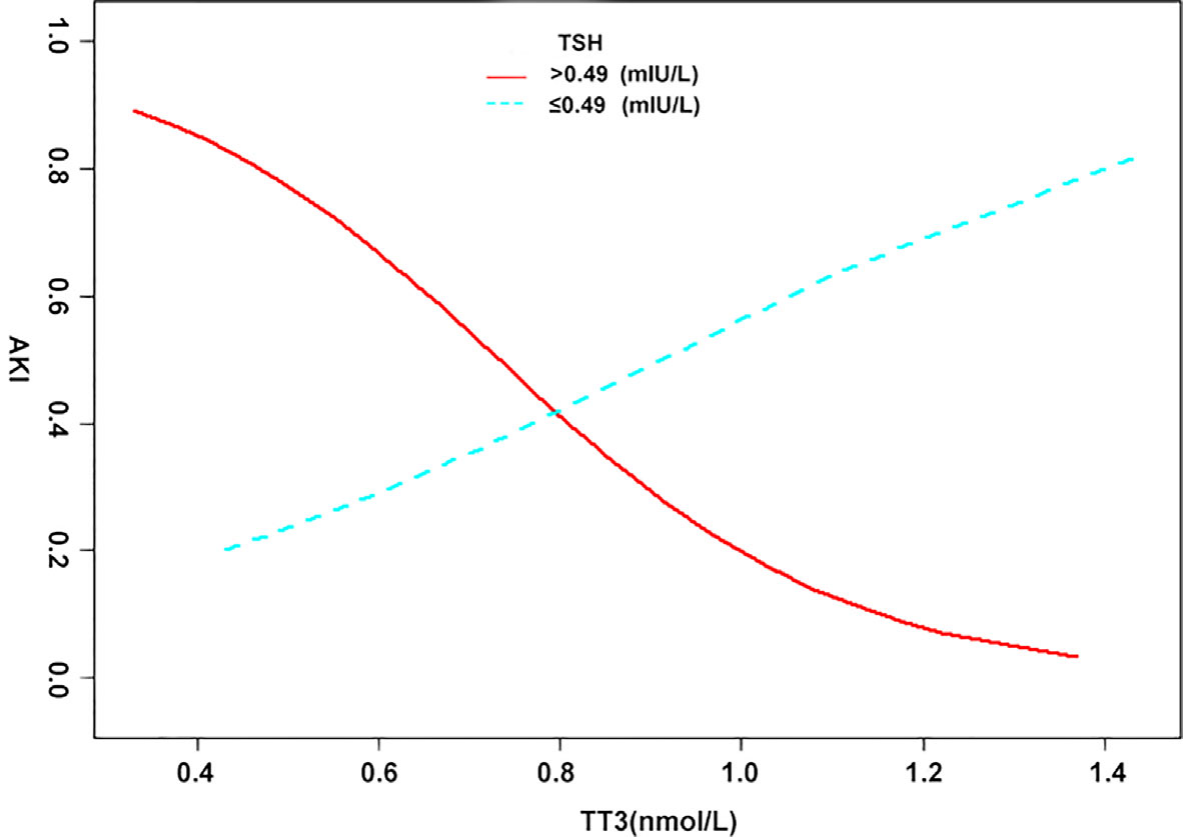
The relationship between thyroid hormone and AKI in different TSH groups. A non-linear association between TT3, TT4, and AKI was found in a generalized additive model (GAM). Solid red line represents the low TSH group. Blue bands represent the normal TSH group. Adjusted for age (years); body mass index (kg/m^2^); platelet (G/L); hematocrit (%); creatinine (μmol/L); intraoperative transfusion of packed red blood cell (u); aortic cross-clamping time (min).

When analyzed as continuous variables, multiple linear regression models showed that postoperative AKI was negatively associated with the preoperative levels of TT3 and TT4 after adjusting for age, sex, BMI, PLT, HCT, creatinine, intraoperative transfusion of PRBCs, circulatory arrest time, and aortic occlusion time (P < 0.05) (**Figure 1**). When patients were classified into the normal TSH group and the lower TSH group, multivariate adjusted smoothing spline plots suggested that the incidence of AKI increased with decreasing TT3 levels in the normal TSH group and increasing TT3 in lower TSH group (**Figure 2**).

## Discussion

In this study, patients with ATAAD had lower preoperative levels of TT3, TSH, and FT3 and higher levels of FT4 than healthy people, indicating that they had conditions such as hypothyroidism. The acute inactivation of thyroid hormones by inner-ring deiodination is probably a beneﬁcial adaptation during NITS, and the common belief is in favor of an attempt by the body to decrease the metabolic rate, suppress systemic energy consumption, and return to baseline as the illness resolves, thereby promoting survival (22). The proportion of ATAAD patients with low T3 levels was as high as 79.5% (70/88), which was similar to a previous report (23). However, the clinical significance of thyroid hormone levels in ATAAD has not yet been confirmed (24).

The relationship between low T3 levels and vulnerable renal function has been observed in patients with chronic clinical hypothyroidism and renal failure (17). The prevalence of subclinical and clinical primary hypothyroidism (low T3) increased with progressively lower levels of kidney function in a nationally representative cohort of U.S. adults (25). The decrease of T3 is also a sign of bad clinical outcomes in uremic patients (26). As a postoperative complication of ATAAD, the occurrence of postoperative AKI is approximately 50% (2, 8). Significant relations between low levels of TT3 and postoperative AKI in patients with ATAAD are shown in the present study. It has been reported that the availability of thyroid hormones influences kidney weight, size, and structure during growth and development (16). Histologic studies have documented the effects of T3 on outer and cortical medullary tubular segments, especially involving the distal convoluted tubule and proximal tubule (26). Some researchers believe that the role of T3 in kidney injury is associated with enhanced epithelial recovery by increasing endogenous epidermal growth factor/epidermal growth factor receptor-mediated renal repair (27). The examination of other etiologic factors has also focused on renal hemodynamics. In fact, clinically overt hypothyroidism (low T3) may cause alterations in renal hemodynamics produced by decreased cardiac output and diminished circulating volume, resulting in a decrease in renal blood flow or ‘prerenal insufficiency’ (28). Furthermore, thyroid hypofunction is related to decreased renin gene expression, decreased sensitivity to beta-adrenergic stimulus and renin release (17), and increased mean arterial pressure, resulting in a reduction in activity of the renin-angiotensin system. This reduction may eventually lead to impaired renal autoregulation (11).

As demonstrated in the subgroup analysis, significant interactions between TSH and TT3 levels with regard to the effect on AKI indicated that the effect of low TT3 levels on AKI was more pronounced within the normal TSH cohort (laboratory results suggest NITS). Based on spline smoothing plots of AKI by TT3 levels, inverse tendencies, albeit not confirmed statistically, were observed in the low TSH groups. From the above results, it is interesting to note that the effects of TT3 levels on AKI were opposite in the two groups. Similar findings have been reported in previous clinical studies. Patients with AKI received intravenous thyroxine or a placebo, but thyroxine had no effect on the course of clinical AKI and may have had a negative influence on the outcome *via* the prolonged suppression of TSH (29). Another study reported that T3 therapy was not beneficial in critically ill patients, and by inhibiting TSH secretion, it may suppress an important mechanism for the normalization of thyroid function during recovery (30). Accordingly, we speculate that exogenous thyroid hormone supplementation suppresses TSH and might increase the incidence of AKI in patients with NITS, even if T3 concentrations rise.

Thyroid hormone supplementation has been demonstrated to ameliorate or reverse ischemic and toxic AKI in a wide variety of animal models (31, 32). Indeed, several studies have reported the use of thyroid hormones in adults in ICU settings with acute non-thyroidal illness (32, 33). Merla et al. found that heart-failure patients with insufficiently treated hypothyroidism have worse renal function than patients whose hypothyroidism is effectively treated. This showed the importance of treating low thyroid levels to improve kidney function in heart failure patients (34).

Nevertheless, the use of thyroid hormones should be approached more cautiously in patients with ATAAD because thyroid hormones might activate the circulatory system, which may increase the possibility of aortic rupture in patients with dissection.

## Limitation

First, because this study is cross-sectional, the present analysis is limited in its capacity to establish causal or temporal relationships between preoperative thyroid hormones and postoperative AKI in ATAAD patients. Second, because all patients underwent CTA, the authors could not exclude AKI caused by contrast-induced nephropathy (CIN), although the incidence of CIN is quite low in patients without renal diseases. Finally, the dynamic data of the effects of thyroid hormones on renal function were not included in the present study.

## Conclusion

In conclusion, the results suggest that low preoperative levels of TT3 and TT4 were independently associated with postoperative AKI in ATAAD patients. Moreover, the effect of low TT3 levels on AKI was relatively more pronounced in the normal TSH group. Therefore, the thyroid function should be checked before surgical intervention of patients with ATAAD, and patients with low T3 might be at higher risk of postoperative AKI.

## Data Availability Statement

The raw data supporting the conclusions of this article will be made available by the authors, without undue reservation.

## Ethics Statement

The studies involving human participants were reviewed and approved by The human study, and the use of human blood were approved by the Ethics Committee of Beijing Anzhen Hospital (Institutional Review Board File 2014019). The patients/participants provided their written informed consent to participate in this study.

## Author Contributions

(I) Conception and design: WJ, HZ, and YZ. (II) Administrative support: YZ and HZ. (III) Provision of study materials or patients: WJ. (IV) Collection and assembly of data: JL and YX. (V) Data analysis and interpretation: JL and YX; All authors contributed to the article and approved the submitted version.

## Funding

This study was supported by the National Key R&D Program of China (NO. 2017YFC1308000), National Natural Science Foundation of China (NO. 81800404), Capital Health Development Research Project (NO. 2018-4-2068), Beijing Municipal Administration of Hospitals’ Youth Program (NO. QML20180601), Foundation of Beijing Outstanding Young Talent Training Program (NO. 2017000021469G254), Beijing Lab for Cardiovascular Precision Medicine, Beijing, China (PXM2020_014226_000017_00377132_FCG) and Key Laboratory of Medical Engineering for Cardiovascular Disease, Ministry of Education.

## Conflict of Interest

The authors declare that the research was conducted in the absence of any commercial or financial relationships that could be construed as a potential conflict of interest.
